# Synthesis and Characterization of Molecular Imprinting Polymer Microspheres of Piperine: Extraction of Piperine from Spiked Urine

**DOI:** 10.1155/2016/5671507

**Published:** 2016-11-27

**Authors:** Rachel Marcella Roland, Showkat Ahmad Bhawani

**Affiliations:** Department of Chemistry, Faculty of Resource Science and Technology, Universiti Malaysia Sarawak (UNIMAS), 94300 Kota Samarahan, Sarawak, Malaysia

## Abstract

Molecularly imprinted polymer (MIP) microspheres for Piperine were synthesized by precipitation polymerization with a noncovalent approach. In this research Piperine was used as a template, acrylic acid as a functional monomer, ethylene glycol dimethacrylate as a cross-linker, and 2,2′-azobisisobutyronitrile (AIBN) as an initiator and acetonitrile as a solvent. The imprinted and nonimprinted polymer particles were characterized by using Fourier transform infrared spectroscopy (FT-IR) and Scanning Electron Microscopy (SEM). The synthesized polymer particles were further evaluated for their rebinding efficiency by batch binding assay. The highly selected imprinted polymer for Piperine was MIP 3 with a composition (molar ratio) of 0.5 : 3 : 8, template : monomer : cross-linker, respectively. The MIP 3 exhibits highest binding capacity (84.94%) as compared to other imprinted and nonimprinted polymers. The extraction efficiency of highly selected imprinted polymer of Piperine from spiked urine was above 80%.

## 1. Introduction

Piperine, a nitrogenous pungent substance, is an alkaloid found in important and oldest spices, namely,* Piper nigrum* (black peppers) and* Piper longum* (long peppers) [1]. It is also known as 1-piperoylpiperidine with the chemical formula of C_17_H_19_NO_3_. Hamrapurkar et al. [2] stated that Piperine is naturally occurring organic compound that belongs to family Piperaceae. The fruits of Piperine possess antidepressant effects, hepatoprotective effects, antioxidant activity, antitumour effects, antibacterial effects, and anticonvulsant effects [2, 3]. Piperine also has the capability of reducing inflammation, relieving pain, improving digestion, and enhancing the bioavailability [4]. Piperine is extensively used in medicinal field for years due to various medicinal properties including painkiller, antioxidant, and bioavailability enhancer.

Molecular Imprinting Technology (MIT) is used to design molecular recognition materials because it is capable of mimicking natural recognition entities like antibodies and biological receptors [5–14]. The original concept of molecular imprinting is developed by Linus Pauling in 1940s, but Wulff and Sarhan stimulated the interest in imprinting materials [6]. According to Vlatakis et al. [15], in the early 1980s, the molecular imprinting polymers (MIPs) were successfully prepared by using noncovalent MIT.

Molecular imprinting is a universal method to produce polymers with high affinity binding sites for organic, inorganic, biological, and chemical molecules or ions. MIPs allow the functional and crosslinking monomers to copolymerize in the presence of the target compound or known as template [16]. Molecular imprinting polymers [17] can be prepared by various methods such as bulk polymerization [18], electropolymerization [19], suspension polymerization [20], emulsion polymerization, two-step polymerization [21], and precipitation polymerization [22]. Zhou et al. [23] mentioned that the controlled/living radical polymerization (CRP) is used to prepare MIP microspheres as it permits more precise control over the molecular weight, composition, and end group functionality of the obtained polymers [24–27]. MIPs show excellent thermal and chemical stability and can be used in aggressive media [15]. According to Yan and Row [28], MIPs have many advantages over their biological counterparts including inexpensive, simple preparation, storage stability, repeated operations without loss of activity, high mechanical strength, durability to heat and pressure, and applicability in harsh chemical media.

Lai et al. [29] stated that MIPs have been used in important application such as chemical sensors [30, 31], capillary electrophoresis and electrochromatography [32], catalysis [33], HPLC stationary phases [34–39], and solid-phase extraction (SPE) [36].

In this research noncovalent imprinting or self-assembly approach is adopted during the course of polymerization. In noncovalent imprinting, the template and a functional monomer interact by noncovalent interactions in the prepolymerization mixture. According to Spivak [40], noncovalent is simpler molecular imprinting method as compared to covalent and semicovalent because it involves synthetic steps toward the prepolymer complex. In this way interaction between the monomer and template is achieved easily when mixed in the solution. This method has been used to produce imprinted polymers of cinnamic acid. In this research as an application these imprinted polymers are used in extraction of Piperine from spiked urine sample.

## 2. Materials and Methods

### 2.1. Materials

Piperine (C_17_H_19_NO_3_) was purchased from Sigma-Aldrich Co. Ltd. (United States), acrylic acid (AA) was bought from Nippon Shokubai Co. Ltd. (Japan), ethylene glycol dimethacrylate (EGDMA) was purchased from Sigma-Aldrich Co. Ltd. (United States), acetonitrile (ACN) was obtained from Kunshan Yalong Trading Co. Ltd. (China), 2, 2′-azobisisobutyronitrile (AIBN) was obtained from Sigma-Aldrich Co. Ltd. (United States), methanol (MeOH) was obtained from Nuasa Kimia Sejati Co. (Indonesia), acetic acid (CH_3_COOH) was purchased from Alpha Chemika (India), potassium bromide (KBr) was obtained from Powder Pack Chem Co. (India), and hexane was obtained from Seidler Chemical Co. (United States).

### 2.2. Equipment

Branson 2510 ultrasonic cleaner was used to disperse the mixtures. Memmert W350T Water Bath-AAR 3060 was used to carry out the polymerization. IR spectra of polymer particles were recorded with Thermo Scientific Nicolet iS10. Scanning electron microscope (JEOL JSM-6390LA) was used to study the morphology of polymer particles. Shaker (NB-101MT) was used to allow the rebinding of polymer particles with template. EBA 20-Hettich was used to centrifuge and separate the polymer particles from the solution. Shimadzu LC-20A, a reversed-phase high performance liquid chromatography (RP-HPLC), was used to evaluate the batch binding of polymer particles.

### 2.3. Synthesis of MIPs and NIP of Piperine

The following procedure was followed during the preparation. 0.5 mmol of template (Piperine), 2 mmol of monomer (AA), 8 mmol of cross-linker (EGDMA), 75 mL of porogen (ACN), and 0.011 g of initiator (AIBN) were added into 150 mL conical flask, respectively. The mixture was sonicated for 10 minutes in order to remove bubbles and allow complete dissolution. Then, the conical flask containing mixture was placed in a bucket of ice cubes and the reaction mixture was purged with nitrogen gas for 15 minutes. Ice cubes were used in this experiment to allow a suitable environment for noncovalent interactions between Piperine and acrylic acid. After that, the conical flask was sealed and placed into a water bath. The polymerization was conducted for 6 hours, initially temperature was maintained at 60°C for the first three hours, and later temperature was raised up to 80°C and maintained for another three hours in order to complete the polymerization. The produced polymer particles were extracted out by using the centrifugation at 5000 rpm for 10 min. The template was removed by washing the MIPs successively in the mixture of methanol and acetic acid (9 : 1, v/v) until the template was not detected by RP-HPLC at 270 nm. The HPLC was conducted by using the C18 column (250 × 4 mm, 5 *μ*m) with the mobile phase consisting of acetonitrile, distilled water, and acetic acid in the ratio of 60 : 39.5 : 0.5, v/v/v, respectively. The flow rate was set at 0.6 mL/min with UV detection at 270 nm and injection volume was set at 20 *μ*L.

The nonimprinted polymeric particles (NIPs) were prepared in the same way without the addition of the template molecule. The similar procedure was used for the synthesis of different molecular imprinted polymers of Piperine with varying composition of AA and EGDMA (Table 1) by precipitation polymerization, namely, MIP 2, MIP 3, and MIP 4 for MIPs as well as NIP.

### 2.4. Batch Binding Assay

A series of 150 mL five conical flasks containing 0.5 g of the MIP (MIP 1, MIP 2, MIP 3, and MIP 4) and NIP beads were added with a 75 mL of acetonitrile containing 0.5 mmol of Piperine. The conical flasks were shaken on the shaker at 100 rpm and the samples were collected at different time intervals (0, 30, 60, 90, 120, 150, 240, and 360 minutes). The collected samples were centrifuged at 5000 rpm for 10 minutes in order to remove any suspended particles and supernatant was used for further analysis. The concentrations of Piperine after adsorption were recorded by using RP-HPLC. The binding capacity of MIPs and NIP of Piperine was calculated [17] by using the following equation:

(1) $$\begin{array}{c} \text{Binding capacity}\left(\;\%\;\right)=\frac{C_{i}-C_{f}}{C_{i}}\times 100, \end{array}$$

where *C*_*i*_ is the initial Piperine concentration in the solution and *C*_*f*_ is the final Piperine concentration in the solution.

### 2.5. Competitive Binding Test

Caffeine was used as a competitive template with the Piperine. A 150 mL conical flask containing 0.5 g of the MIP 3 beads was added and a solution of 75 mL of acetonitrile containing equal concentration (0.5 mmol) of Piperine and Caffeine. Similarly, for NIP same procedure was followed. Both of the conical flasks were shaken on the shaker at 100 rpm and the samples were collected at different time intervals (0, 30, 60, 90, 120, 150, 240, and 360 minutes). After shaking at the appropriate time intervals, all the samples were centrifuged at 5000 rpm for 10 minutes. The binding of both the templates was monitored by using RP-HPLC. The distribution ratios (mL g^−1^) of Piperine between the MIPs or NIP in the porogen (acetonitrile) were determined [41] by the following equation:

(2) $$\begin{array}{c} \text{Distribution ratio: }K_{D}=\frac{\left(C_{i}-C_{f}\right)V}{C_{i}m}, \end{array}$$

where *C*_*i*_ is the initial Piperine concentration in the solution, *C*_*f*_ is the final Piperine concentration in the solution, *V* is the volume of porogen (ACN) used, and *m* is the mass of MIP/NIP used.

The selectivity coefficients for Piperine relative to binding competitor Caffeine for MIP 3 and NIP can be calculated [41] by

(3) $$\begin{array}{c} \text{Selectivity coefficient: }K_{\text{Piperine/Caffeine}}^{\text{sel}}=\frac{K_{D}^{\text{Piperine}}}{K_{D}^{\text{Caffeine}}}, \end{array}$$

where *K*_*D*_^Piperine^ is the batch binding assay of MIP/NIP for Piperine and *K*_*D*_^Caffeine^ is the batch binding assay of MIP/NIP for Caffeine.

The relative selectivity coefficient (*k*′) was determined [41] by the following equation:

(4) $$\begin{array}{c} k^{\prime }=\frac{k\left(MIP\;3\right)}{k\left(NIP\right)}. \end{array}$$

### 2.6. Extraction of Piperine from Spiked Human Urine

About 100 mL of fresh urine was collected from a drug free human. Urine sample was first centrifuged and filtered and then spiked with a Piperine to get a concentration of 50 ppm. After this 50 mL of spiked urine sample was added in flask containing 0.5 g of MIP 3. The samples were collected and analysed the same as followed in batch bind assay. The NIP was treated in the same way.

## 3. Results and Discussion

Synthesis of microsphere imprinted polymers is a very crucial step in order to produce uniform shape and size of particles. Previous studies revealed [42] that various preparation methods have been carried out for the preparation of polymer microspheres [43] such as the synthesis of polymer microspheres by dispersion and emulsion polymerization, where the surfactants in aqueous solution [44] and stabilizers in organic solution [45] are crucial to stabilize the polymer phase and prevent the aggregation of particles. Precipitation polymerization can form polymer microspheres with constant size and shape that can lead to narrow dispersion, without the need for any added surfactant or stabilizer [46–49]. In this research we have successfully produced imprinted polymer microspheres by precipitation method in the acetonitrile (porogen).

### 3.1. Fourier Transform Infrared Spectroscopy (FT-IR)

**Figure 1:**
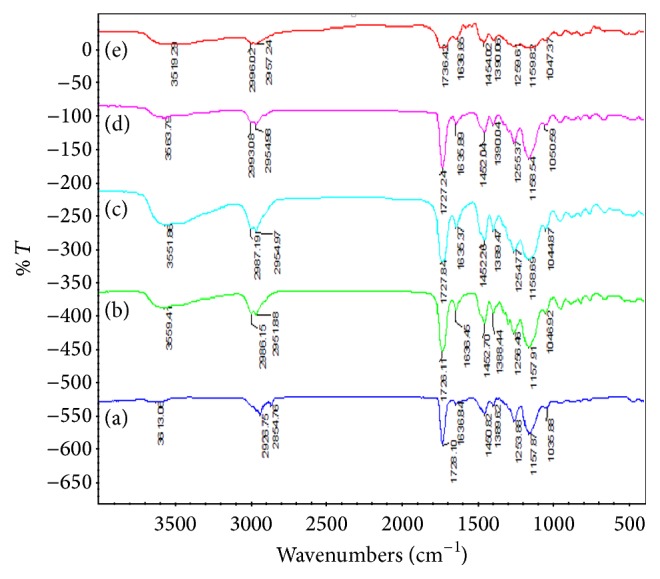
FT-IR spectra, (a) MIP 1, (b) MIP 2, (c) MIP 3, (d) MIP 4, and (e) NIP.

IR analysis is an important chemical characterization method to detect the functional groups present in a compound. The FT-IR spectra of different MIPs and NIP are shown in Figure 1.

Based on Figure 1, small peak in the range of 3519.29 cm^−1^ to 3613.06 cm^−1^ attributed to the vibration mode of O–H stretching was observed in both MIPs and NIP. The bands in the range of 2926.75 cm^−1^ to 2996.02 cm^−1^ and 2854.76 cm^−1^ to 2957.24 cm^−1^ showed the vibration mode of C–H stretching of aliphatic compound as well as asymmetric and symmetric CH_2_ stretching in MIPs and NIP. Strong peaks at 1726.11 cm^−1^ to 1736.42 cm^−1^ indicated the presence of C=O of acrylic acid. The vibration mode of C=C stretching of aromatic compound can be found within 1635.37 cm^−1^ to 1636.84 cm^−1^. The CH_2_ bending at 1450.82 cm^−1^ to 1452.70 cm^−1^ indicated the presence of alkane group in MIPs and NIP.

The vibration mode of C–N stretching of MIPs and NIPs after leaching was detected at 1388.44 cm^−1^ to 1390.06 cm^−1^. Peaks of MIPs and NIP at 1253.88 cm^−1^ to 1259.61 cm^−1^ showed the vibration mode of –O–CH_2_–O– symmetric stretching. Small band at 1035.88 cm^−1^ to 1050.59 cm^−1^ in MIPs and NIP explained the presence of symmetric stretching of =C–O–C. The vibration mode of C–H stretching of aliphatic compound can be observed at 2954.09 cm^−1^ to 2985.19 cm^−1^.

### 3.2. Scanning Electron Microscopy (SEM)

**Figure 2:**
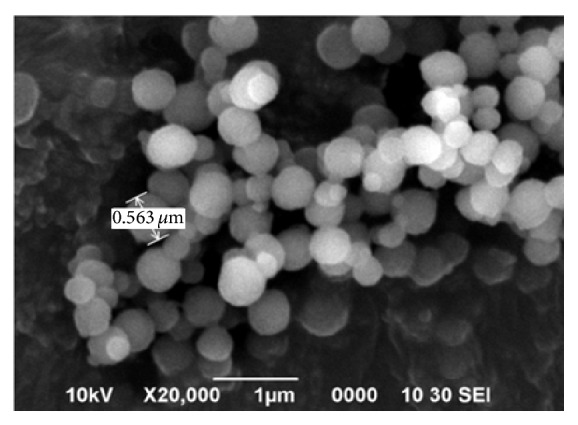
SEM of imprinted polymer particles.

SEM analysis is a very important morphological study for polymer particles that provides the idea about the shape and size.  Figure 2 clearly indicates that spherical particles are produced with the size in micrometres. This is because the polymer particles were synthesized by precipitation polymerization. According to Tamayo et al. [50], uniform size of imprinted polymers can be formed by using a noncovalent imprinting approach by precipitation polymerization.

Arabzadeh and Abdouss [41] stated that interaction between monomer and template could be another factor that contributed to uniform size distribution with clean surfaces. Research conducted by Park et al. [51] mentioned that there are various factors that affect the production of uniform polymer microspheres including volume of solvent, reaction of solvent, and presence of template ion. Excess solvent or porogen used in the synthesis of polymer particles will produce highly uniform polymer microspheres with imprinted binding sites.

### 3.3. Batch Binding Assay of MIPs and NIP

**Figure 3:**
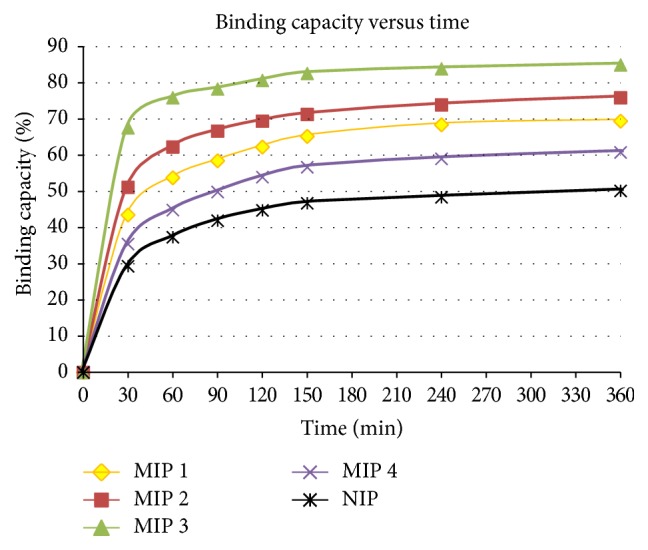
A graph of binding capacity of MIPs 1, 2, 3, and 4 and NIP at different time intervals.

RP-HPLC was used to evaluate the binding efficiency of MIPs and NIP of Piperine.  Figure 3 depicts the binding capacity of different MIPs and NIP at different time intervals.

MIP 3 showed the highest binding capacity (84.94%), followed by MIP 2 (75.86%), MIP 1 (69.40%), and MIP 4 (60.80%). MIP 3 contains a higher amount of monomer ratio as compared to MIP 1 and MIP 2 but MIP 4 contains a higher amount of cross-linker. In this study increasing amount of monomer would produce specific interaction sites with the Piperine and hence rebinding efficiency was also increased. But the increase in amount of cross-linker has produced a reverse effect as can be seen in MIP 4. If we compare the MIPs with NIP it is clear from Figure 3 the binding capacity is low. This can be conferred that NIP does not contain any binding site complimentary with the Piperine.

### 3.4. Competitive Binding Assay

In order to evaluate the properties of MIP of Piperine as a sensing material, the selectivity test was conducted. In this test, two compounds (Piperine and Caffeine) were tested using both MIP 3 and NIP. The selectivity of Piperine and Caffeine was calculated by using RP-HPLC measurements. The distribution ratio of Piperine in both MIP 3 and NIP was higher than the distribution ratio of Caffeine in both MIP 3 and NIP, resulting in higher selectivity coefficient of Piperine than that of Caffeine in both MIP 3 and NIP (Table 2). The results indicate that the imprinted polymer has got complimentary binding sites or cavities with the Piperine as compared to Caffeine.

### 3.5. Extraction of Piperine from Spiked Human Urine

The extensive use of Piperine in medicine and spices has generated this idea to first use the selected MIP 3 in the extraction of Piperine from urine. This will provide us a way forward to expand the application of these imprinted polymer particles. From this study it was found that about 81.18% of Piperine was successfully extracted from the spiked urine sample.

## 4. Conclusion

Molecularly imprinted polymeric microspheres of Piperine were synthesized by using precipitation polymerization. The binding efficiencies of MIPs and NIP of Piperine were evaluated by batch binding assay. MIP 3 exhibited the highest binding capacity (84.94%) as compared to NIP (40%). These imprinted polymer particles successfully extracted (81.18%) Piperine from spiked urine.

## Figures and Tables

**Table 1:** Synthesis of molecularly imprinted polymers and nonimprinted polymer for Piperine by precipitation polymerization.

Code	MIP 1	MIP 2	MIP 3	MIP 4	NIP
Template (mmol)	Piperine (0.5)	Piperine (0.5)	Piperine (0.5)	Piperine (0.5)	—
Monomer (mmol)	AA (2)	AA (2)	AA (3)	AA (3)	AA (3)
Cross-linker (mmol)	EGDMA (8)	EGDMA (12)	EGDMA (8)	EGDMA (12)	EGDMA (8)
Porogen (mL)	ACN (75)	ACN (75)	ACN (75)	ACN (75)	ACN (75)

**Table 2:** The distribution ratio, selectivity coefficients, and relative selectivity coefficient of MIP 3 and NIP.

	*K*_*D*_ (MIP 3) (mL g^−1^)	*K*_*D*_ (NIP) (mL g^−1^)	*k* ^sel^	*k*′
Piperine	76.72	26.79	5.34	—
Caffeine	14.37	7.70	3.47	1.54
